# Postmortem forensic toxicology cases: A retrospective review from Milan, Italy

**DOI:** 10.1111/1556-4029.15050

**Published:** 2022-05-04

**Authors:** Domenico Di Candia, Gaia Giordano, Michele Boracchi, Riccardo Zoja

**Affiliations:** ^1^ Sezione Di Medicina Legale, Dipartimento Di Scienze Biomediche per La Salute Università Degli Studi Di Milano Milan Italy

**Keywords:** acute intoxication, autopsy, forensic toxicology, homicide, retrospective study, suicide

## Abstract

We are presenting a study on 136 cases performed in a 2‐year period (2018–2019) at the Bureau of Legal Medicine of the University of Milan for which toxicological analyses were requested and we are making a detailed interpretation of clinical records and discussing toxicological results from each case included in the study. Total number of autopsies was 1323 and in 10.3% of the cases, toxicological analyses were requested to obtain further information. Analyses were assessed with High‐Performance Liquid Chromatography‐Mass Spectrometry system and Gas Chromatography–Mass Spectrometry analyses. Additionally, Blood Alcohol Concentration and detection of volatile substances were obtained with Head Space‐Gas Chromatography–Mass Spectrometry system. From these analyses, 101 cases out of 136 provided positive results (74.3%). Main substances detected were cocaine, diazepam, morphine, and ethanol. The most representative profiles of individuals that emerged from this study were: a Caucasian male, age 41–50, that died for cocaine acute intoxication or was killed; a Caucasian male or female with a range‐of‐age of 31–50 deceased for simple suicide caused by acute intoxication or by complex suicide caused by acute intoxication and suffocation; and finally, a Caucasian male with a range‐of‐age 21–40 that died in a car accident without any toxicological evidence. From the results, acute intoxication at the time of death was confirmed in 54 cases and in 57 cases the toxicological analyses helped in the determination of the cause and manner of death. From this study, the importance of toxicological data among forensic sciences is confirmed.

**Highlights**

- Toxicological analyses in a 2‐year period.
- Drug related fatalities, suicides, homicides on 136 toxicological cases.
- The most common individual was a Caucasian male age 41–50 years.
- Acute intoxication was observed in 39.7% of the investigated cases.
- The most common drugs detected were cocaine, ethanol, diazepam, and morphine.

## 1 | INTRODUCTION

Forensic toxicology is a branch of toxicology at the service of forensic sciences [1, 2]. It consists in compounds analyses, focusing on the interpretation of results in a medico‐legal context [1]. In forensic toxicology, qualitative and quantitative analyses are performed with the objective of interpreting the likely role of a compound in a case under investigation. Indeed, the forensic toxicologist contributes to establish both causes and modalities of either intoxication or death through the analysis of different fluids and tissues obtained from autopsy examination [1]. Some studies already discussed the causes and manner of death of individuals in Italy through records papers, generally focusing on simple, complex or complicated suicides. For example, Pelletti et al. [3] made a study at the Bureau of Legal Medicine of Bologna from 2013 to 2017 reporting the increasement of prescription drugs in complex suicides. Another study, analyzed all the fatalities of an Italian region (Friuli‐Venezia Giulia) from 1963 to 2017 focusing on complex suicides and finding a total of eight cases of Plastic Bag Suffocation (PBS) combined with gas inhalation and 4 male individuals that committed planned complex suicides with firearms, however the toxicological findings were not considered in this study [4]. Barranco et al. [5], from the Bureau of Legal Medicine of Genoa, selected complex and complicated suicides from 2006 to 2017, finding one death of complex suicide performed with a combination between benzodiazepines, alcohol and wrist cutting. Moreover, from a Literature review emerged one study, achieved at the Bureau of Legal Medicine of Parma, that collected, from 2009 to 2016, 1005 cases [6]. The authors of this study noted that in most deaths more than one drug was involved (precisely in 75.4%, or 758 cases); heroin or morphine were combined with other central nervous system depressant drugs (e.g., alcohol in the 42.0% of the cases or prescription drugs in about 88.0%). In general, Anzillotti et al. [6] highlighted heroin injection as the major cause of death in acute intoxication cases and the substance of abuse most detected was alcohol whereas the second one morphine.

In this study, we examined all the toxicological analyses requested on the autopsies performed at the Bureau of Legal Medicine of the University of Milan from January 2018 to December 2019. Generally, the conventional biological specimens that can be collected from cadavers in order to perform toxicological analyses are blood samples (femoral, cardiac or thoracic blood), urine, vitreous humor, gastric content, organs (manly liver and lung) [7]. From toxicological analyses and data collection we created a database containing, for each case under investigation, sex, age, ethnicity, nationality of the individuals; moreover, clinical history, manner and cause of death were reported (in accordance with ISTAT (*Istituto Nazionale di Statistica* [8] data), if present, and the results of toxicological analyses were noted (together with the matrices analyzed, the substances and the concentration obtained from the analyses).

At the Bureau of Legal Medicine of the University of Milan other records were already performed, overviews on suicides and natural deaths in prison [9], suicides [10, 11], and homicides [11, 12], but none of them reports a study on toxicological findings obtained from forensic investigations performed on corpses.

Therefore, in this paper, we present a retrospective study of the toxicological analyses performed at the Bureau of Legal Medicine of the University of Milan from 2018 to 2019. From the toxicological analyses, requested for further investigations by the Judicial Authority, performed on deceased that arrived at the Bureau of Legal Medicine of the University of Milan, we created a wide and rich database of 136 toxicological analyses on a 2‐year period.

## 2 | MATERIAL AND METHODS

Autopsy examinations performed at the Bureau of Legal Medicine of the University of Milan from 2018 to 2019 were 1323. From the total number of cases, we selected the individuals for which the toxicological analyses were requested by the Judicial Authority in order to clarify the cause and manner of death of the subjects, thus obtaining 136 subjects. In this paper were reported sex, age, ethnicity, clinical history, toxicological findings, cause and manner of death of the subject under investigation.

The analyses were performed on different biological samples, mainly on femoral and cardiac blood, urine and gastric content, as routine practice of the laboratory. In some cases, when conventional matrices were not available or when the case required further investigation on different matrices, some organs were analyzed (e.g., liver, brain, or kidney) (Table S1). Detection and quantitation of substances on femoral blood are considered as gold standard for the determination of a state of intoxication of an individual; cardiac blood was selected as control sample of femoral blood or in absence of other standard hematic matrices; urine and gastric content were analyzed for completeness of toxicological investigations. The ratio between cardiac and femoral blood in drug related and non‐drug related deaths was 0.99, with a minimal ratio for clozapine at 0.4 and with a maximal ratio for ketamine at 1.7 (Table 1).

**TABLE 1:** The table reports the ranges and average of post‐mortem blood concentrations in drug‐related deaths and non‐drug related death caused by a single substance. Concentrations are reported in μg/ml, if not the correct unit of measurement is reported in table

		Drug detected	n. of cases	Range of cardiac blood	Average of cardiac blood	Range of femoral blood	Average of femoral blood	Ratio cardiac blood on femoral blood
Drug related death by single substance	Simple suicide	Cyanide	1	/	/	/	/	/
	Complex suicide	Cocaine	1	1.53	/	1.02	/	1.5
	Acute intoxication	Cocaine	15	0.33–19.8	5.37^a^	0.49–18.78	5.28^b^	1.0
		Phenobarbital	1	96.20	/	108.03	/	0.9
		Methadone	3	1.0–6.45^c^	3.72	1.1–9.22	3.8	1.0
		Free Morphine	4	1.98–2.84	1.99	1.67–4.89	2.69	0.7
		Ketamine	1	5.69	/	3.36	/	1.7
		Quetiapine	1	/	/	9.16	/	/
		Sertraline	1	2.34	/	2.13	/	1.1
Non‐drug related death by single substance	Complex suicide	Cocaine	1	0.09	/	0.14	/	0.6
	Simple suicide	Clozapine	1	0.1	/	0.24	/	0.4
		Fluoxetine	1	/	/	Traces^d^	/	/
	Heart failure	Citalopram	1	Traces^e^	/	/	/	/
		Diazepam	1	2.31 ng/ml	/	2.03 ng/ml	/	1.1
		Oxycodone	1	16.4 ng/ml	/	22 ng/ml	/	0.7
	Car accident	Midazolam	1	0.09	/	0.08	/	1.1

^a^Average calculated on 13 cases, considering that in two individuals (nos. 35 and 61) the cardiac blood was missing. ^b^Average calculated on 14 cases due to the fact that in one case (no. 35) the femoral blood was missing. ^c^Average calculated on 2 cases, considering that in one case (no. 93) the femoral blood was missing. ^d^Lower than LLOQ: less than 5 ng/ml. ^e^Lower than LLOQ: less than 1 ng/ml.

All the biological samples were collected from cadavers during autopsy examination performed at the Bureau of Legal Medicine of the University of Milan and were stored in a freezer at −20°C. Toxicological analyses may be requested by the Judicial Authority for further investigations. Toxicological analyses were carried out following the protocols of the Laboratory of Forensic Toxicology of the University of Milan. Analyses for molecules of toxicological interest were assessed with High‐Performance Liquid Chromatography–Tandem Mass Spectrometry system TSQ Fortis II, as reported in ref. [13], and Gas Chromatography–Mass Spectrometry instrument 5890 Agilent Technologies with a Mass Selective Detector 5975, as mentioned in ref. [14], whereas Blood Alcohol Concentration (BAC) and the detection of volatile substances were performed with Head Space‐Solid Phase Micro Extraction using a Gas Chromatography–Mass Spectrometry (Thermo Fisher Scientific, DSQII), as reported in a previous study [13]. All samples were previously screened with a NIST library and, when matching, molecules were added to a customized inclusion list and samples were run again in order to perform a qualitative analysis. If confirmed, substances were quantitated after setting up appropriate calibration curves starting from appropriate working standards solutions.

Limit of detection, signal‐to‐noise ratio (S/N) of at least 3, and Lowest Limit of Quantification, S/N ≥ 10, of each substance are reported in Table S2.

## 3 | RESULTS

In these 2 years, toxicological analyses were required by the Judicial Authority on 136 cases (10.3% of the total number of autopsies), in which there were 33 females (23.9%) and 103 males (75.7%) (Tables S1 and S3). The majority of the individuals' ethnicity was represented by Caucasian (131–96.3%), followed by two Latin Americans, two Asians and one of African ethnicity (Tables S1 and S3). The deceased were most frequently Italians (110–80.9%). Furthermore, the presence of five Moroccans and two Tunisians was noted, as well as the presence of an Algerian, a Peruvian, a Colombian, a French, a German, an Albanian, a Filipino, a Brazilian, a Turk, a Belgian and a Sri Lankan. Additionally, individuals were divided in nine range‐of‐age groups: <1 year (with three individuals, average of 4 months ± 1.4), 1–10 (none of the cases were present in this category), 11–20 (with four representants, average 18 years of age ± 3.4), 21–30 (24 individuals, average of age 25.3 ± 3.0), 31–40 (23 individuals, average of age 35.8 ± 3.0), 41–50 (34 cases, average 46 years of age ± 2.5), 51–60 (20 individuals with an average of age 54.8 ± 2.9), 61–70 (10 representants, average of age 65.9 ± 2.1), 71–80 (4 cases, average of 74.3 years ±4.0) and 81–90 (with six cases and an average of age 83.5 ± 1.2).

Therefore, the age of the individuals that arrived at the Bureau of Legal Medicine of the University of Milan for autopsy examinations ranged from 3 months to 85 years old and the most represented range‐of‐age was 41–50 in both genders (Table S3). The most represented individual is a Caucasian male from 41 to 50 years of age.

**FIGURE 1:**
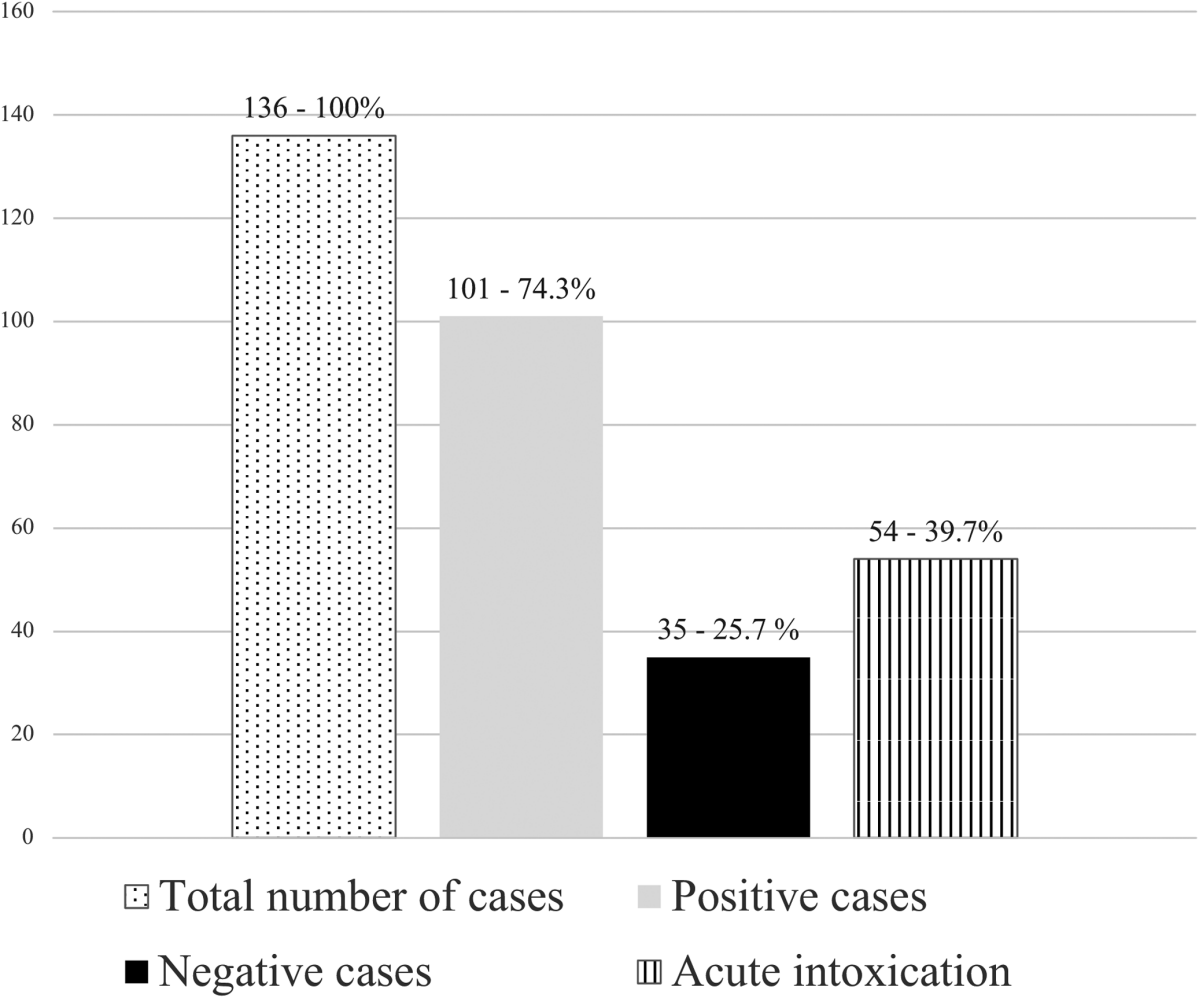
Graphic representation of toxicological analyses performed from the 2018 to the 2020 at the Bureau of Legal Medicine of Milan. In table are represented in “first column” the total number of cases investigated in the 2 years under examination, in “second column” the positive results, in “third column” the negative results and in “fourth column” the number of cases, compared with the total number of toxicological cases analyzed (136), in which the cause of death was due to “acute intoxication” of some prescription drugs or illicit drugs.

From these analyses, 101 cases provided positive results (74.3% of the total number of cases in which the toxicological investigation was requested) and, as a consequence, 25.7% (or 35 cases) of the cases under investigation gave negative results (Figure 1 and Table S3).

Moreover, in 54 cases the cause of death was ascertained as “fatal acute intoxication” thanks to the toxicological analyses performed (39.7% of the total number of cases investigated in this study and the 53.5% of the positive cases) (Figure 1 and Table S3).

### 3.1 | Positive toxicological findings

From analyses, several drugs and illicit substances were detected. Table S2 reports the number of cases in which molecules, and their metabolites, were detected; the percentage of cases compared to the total number of cases (136) and to the number of positive results (101) in investigated matrices. Dividing the positive results per sex, 74 out of 103 males (71.8%) were positive to some substances and 26 out of 33 females (78.8%) had positive toxicological results.

The most representative molecules, and metabolites, detected were several: benzoylecgonine, the metabolite of cocaine, (detected in 38 cases, which means 27.9% of the total number of cases and 37.6% in respect with the positive results obtained in 101 cases); cocaine (found in 37 cases, 27.2% of the total number of cases and 36.6% of the cases that gave positive results); ethanol was detected in 36 out of 136 cases (26.5%) and corresponding to 35.6% (36 out of 101) of the individuals with positive results; diazepam, a benzodiazepine, was identified in 22 cases, which means 16.1% and 21.8% of total number of cases and of positive cases only, respectively; morphine was detected in 13 cases, obtaining the 9.5% of detection on total number of cases and the 12.8% on the cases in which positive results were provided. Other drugs and illicit substances detected in less than 10 cases were listed in detail in Table S2.

From now on we are referring to “acute intoxication” for the cases in which xenobiotics were considered as the cause of death by the forensic practitioner on the bases of toxicological results, circumstantial data, and autopsy findings. For the cases of complex suicide, we are still using the terms “acute intoxication” because if main detrimental non‐toxicological mean was not defined as the cause of death, the concentration of xenobiotics quantitated in biological matrices could have led in a longer period to the deceasing.

### 3.2 | Substances and concentrations detected in acute intoxication cases

Tables 1 and 2 show the cases in which the cause of death consisted in an acute intoxication. Table 1 details the concentrations obtained in cardiac and femoral blood, whereas in Table 2 are listed the concentrations detected in the other matrices analyzed. In order to provide a complete overview of all the matrices involved in the study, we have summarized the results obtained in all the matrices different than blood in Table 2. A total amount of 54 cases of acute intoxication were found (53.5% of the positive cases and the 39.7% of the total number of cases under investigation), of which 28 were caused by the administration of a single drug. Furthermore, 39 out of 103 (37.8%) males and 15 out of 33 (45.5%) females died of acute intoxication, considering as a population the total number of males and females analyzed (both positive and negative results).

**TABLE 2:** The table lists the drugs and illicit substances that led to acute intoxication. The cases considered are the subjects deceased for acute intoxication caused by a single drug (μg/ml)

Drug	N° of cases	Mean (range) of urine	Mean (range) of gastric content	Mean (range) of bile	Mean (range) of liver	Average of liver	Mean (range) of spleen	Mean (range) of brain	Mean (range) of lung	Mean (range) of kidney	Mean (range) of fat	Mean (range) of gallbladder
Cyanide	1 (1.8%)	/	/	/	/	/	/	/	/	/	/	/
Cocaine	16 (29.6%)	8.36 (0.43–42.06)	162.65 (1.07–1127.0)	3.17 (0.33–4.78)	0.03–8.83	2.98	/	5.76	/	/	/	/
Phenobarbital	1 (1.8%)	128.38	988.91	/	/		/	/	/	/	/	/
Methadone	3 (5.4%)	54.72	0.49 (0.32–0.658)	561	8.31		/	/	/	/	/	/
Free Morphine	4 (7.4%)	17.8 (4.2–33.83)	7.42 (0.02–29.09)	/	0.43		0.37	/	/	0.65	/	1.15
Ketamine	1 (1.8%)	18.27	39.44	/	/		/	/	/	/	/	/
Quetiapine	1 (1.8%)	37.97	4611.3	/	/		/	/	/	/	/	/
Sertraline	1 (1.8%)	0.07	34.94	/	/		/	/	/	/	/	/

The illicit drug responsible for most cases of acute intoxication with 16 deaths (29.6%) (one of them was a complex suicide), was cocaine, followed by morphine (4 cases – 7.4%) and methadone (3 cases – 5.4%) (Table 2).

Moreover, some cases of acute intoxication were characterized by the combination of some substances together: amlodipine combined with alprazolam (1 case); cocaine and morphine (4–7.4% of the total number of acute intoxication); cocaine and methadone (1 case); cocaine and methomyl (1 case); cocaine and MDMA (1 case); cocaine and paroxetine (1 case); cocaine and sertraline (1 case); cocaine and MDPHP (1 case); delorazepam together with bromazepam (1 case); delorazepam with alprazolam (1 case); olanzapine together with paroxetine (1 case); diazepam and lorazepam (1 case); oxycodone combined with bromazepam and lormetazepam (1 case); diazepam, bromazepam and lormetazepam (1 case); alprazolam, tramadol, quetiapine and venlafaxine (1 case); clozapine combined with alprazolam, diazepam, paroxetine, olanzapine, pregabalin, clonazepam, lorazepam and delorazepam (1 case).

### 3.3 | Negative toxicological findings

**FIGURE 2:**
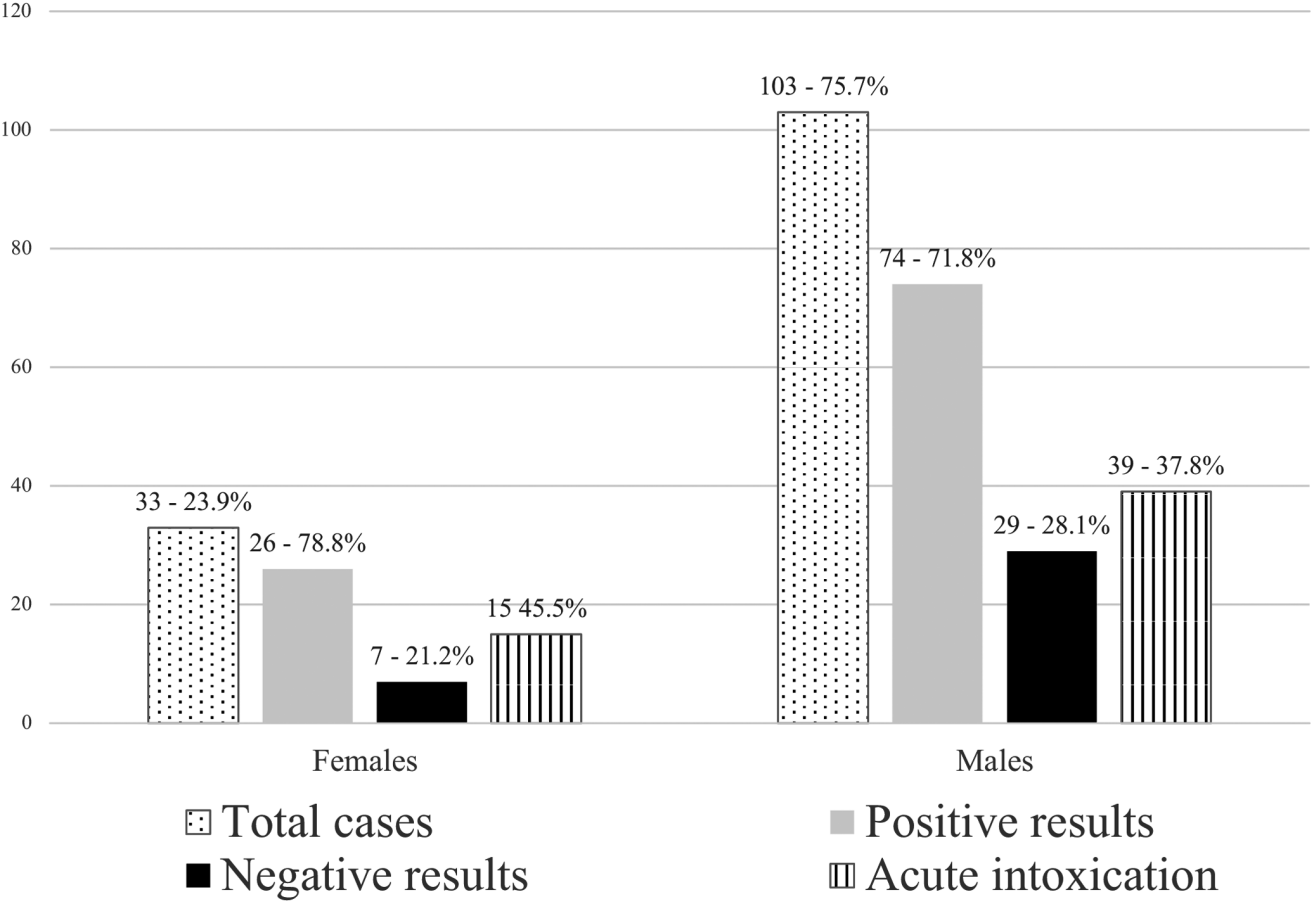
Positive results and acute intoxication cases in females and males’ groups; in “first column” the total number of females and males' cases; in “second column” the total number of positive results; in “third column” the negative results; in “fourth column” the cases in which the acute intoxication was the cause of death.

Thirty‐five negative results were detected from the 136 cases considered between 2018 and 2019. The population of 35 individuals was composed by 28 males and 7 females, that is 80.0% and 20.0% of males and females, respectively. Moreover, 27.2% (28 out of 103) of males and 21.2% of females (7 out of 33) did not take drugs and illicit substances, included in the scope of testing performed, close to the time of death (Figures 1 and 2).

### 3.4 | Clinical history, cause, and manner of death

Table S3 reports the clinical history, the cause and manner of death of each case from the data collection and toxicological analyses. Evaluating the clinical history of the individuals (clinical history was recorded from relatives’ interviews, clinical records, first responders’ information or eyewitnesses), 10 were suffering from drug addiction, two were suspected of being drug addicted, two had a history of drug addiction in the past and one already had experienced a non‐lethal acute intoxication by heroin. Additionally, 13 individuals were suffering from major depression disorder, one had major depression disorder and had reported suicide thoughts before death, five had psychiatric diseases (mainly schizophrenia and borderline personality disorder) and one had judgment inabilities. Moreover, two individuals suffered from alcoholism, three were subjected to involuntary medical treatment and one was hospitalized for major depressive disorder. Furthermore, four individuals previously attempted to commit suicide, one was in drug rehabilitation, and one had attended a drug addiction recovery program.

The data collection (recorded from relatives’ interviews, clinical records, first responders’ information or eyewitnesses) allowed to highlight cause and manner of death, provided by the forensic pathologist, in many cases: in four cases the cause of death was accidental (two by fall, one was a work accident, and one did not report circumstantial data), in one case was beaten to death, in 13 cases the manner of death was consequent to car accidents, whereas in one case a man was hit by a car. In addition, in one case the cause of death was caused by a cold weapon, in two cases by shot guns, and in one case a man died for second‐degree murder. Furthermore, in 46 cases the cause of death was acute intoxication (of which one was an infanticide), while in three cases the suicide was induced by acute intoxication. Moreover, we documented that suicide was committed by fall in five cases and in five cases by suicide (by volatile inhalation or external suffocation) was combined with acute intoxication; whereas in one case the individual committed suicide by hanging. Additionally, in one case the suicide was reported in clinical records without circumstantial data (Table S3).

### 3.5 | Accidental death: Car accidents

Thirteen individuals died because of a car accident, in which seven of them had negative results to toxicological findings. In four cases, individuals were under the effect of alcohol with concentrations that varied from 0.83 to 1.38 g/L. With reference to the Italian traffic laws, a BAC above 0.5 g/L entails the inability of a person to drive. The effects of alcohol on the human body increases with the increasing of the BAC: from 0.5 to 1.0 g/L the effects on an occasional drinker are euphoria, major socialization, motor incoordination; instead from 1.0 to 2.0 g/L, the effects are dysarthria, ataxia, drowsiness, emotional instability, and nausea. Even though an alcoholic (an individual to whom has been diagnosed an alcohol abuse disorder) has minimal or null effects with a BAC of 0.5 to 1.0 g/L, he has motor incoordination from 1.0 to 2.0 g/L [15].

The presence of midazolam in one case (case no. 7; male, 21–30 years) and the presence of midazolam and ketamine in another one (case no. 123; male, 14–50 years) can be referred to a hospital administration as sedative and for severe pain treatment in case of polytraumatic events (Table S3).

### 3.6 | Accidental death: Fall and work accident

Other four cases of accidental death were recorded in the archives of the Bureau of Legal Medicine of the University of Milan, one of them was a work accident of a male, in the range‐of‐age 21–30 years with no positive toxicological results, while the other three individuals died for accidental death: the first one (case no. 101) was a female fallen while cleaning the windows of her home; the second one was a young male in which LSD concentration was in accordance with an altered psychomotor state with visual and sensory alterations sufficient to determine a distorted perception of reality of the subject; the last was a precipitated man in which the condition of accidental death was dictated by the circumstantial data, the autopsy carried out by the medico‐legal practitioner and the toxicological results which reported the presence, in traces, of venlafaxine in the urine sample only (Table S3).

### 3.7 | Accidental death: Acute intoxication

From the study of the data, it can be noted that the causes of death due to acute intoxication remain stable throughout the years evaluated. The total number of deaths caused by acute intoxication were 54: precisely, 46 involuntary acute intoxication, 3 suicide via acute intoxication by some substances and 5 complex suicides in which the acute intoxication had a role in the cause of death.

Indeed, the death in complex suicides may have occurred due to another mean (e.g., case no. 20, cause of death: acute intoxication by butane and PBS) than the substances administration.

These data are in accordance with other Italian records, based on toxicological analyses [6], in which the accidental death caused by substances are the most represented followed by drug intake suicides.

Moreover, during 2018, 30 individuals died due to acute intoxication, as well as in 2019 24 individuals died from the same cause.

From data collected during this study it was highlighted that 78.8% (26 out of 33 cases) of females were positive to some prescription drugs or drugs of abuse and in 45.5% of the cases (15 cases) the women died for acute intoxication, while 71.8% of males (74 out of 103 cases) had positive findings to toxicological analyses; of whom in 39 cases (37.8%) death was due to acute intoxication. Only 21.2% and 28.1% of females and males respectively, were negative to some toxicological findings (Figure 2). Moreover, 21 individuals, included in the range‐of‐age 41–50, died by acute intoxication.

The most common place of corpse discovery was individual's home. As a matter of fact, bodies were discovered in that location 14 times (7.6%, that is 14 out of 54 acute intoxication cases). Additionally, in four cases bodies were found in a park, two times in the car of the deceased, two times in a hotel room and once in jail. Only in two cases subjects were found still alive and transported to the hospital but died later. However, in 22 cases the circumstantial data did not report where the body was found (Table S3).

Cocaine was the illicit substance mostly detected (Table S1), responsible for 31.5% of the acute intoxications, and it was the single molecule that caused acute intoxication in 15 cases. In two cases, this substance was used for combined suicides. In these 17 cases, 76.5% of the individuals were male, while the remaining 23.5% were females with a range‐of‐age of 41–50 years for both genders.

Moreover, it was noted that the number of cases of cocaine intoxication decreased of about 45.5% from 2018 to 2019. In fact, the number of deaths passed from 11 to 6 from 1 year to another.

As reported in literature [16, 17, 18], it should be noticed that cocaine combined with other drugs induced the death in 11 cases. In four cases, cocaine was combined with morphine (leading to death), while in one case it was combine with morphine and ethanol. In the remaining six cases, cocaine was combined with methomyl, MDPHP, sertraline, methadone, MDMA and paroxetine, respectively (Table S3). However, evaluating the Italian overview, Anzillotti et al. [6], declare that the substance most frequently involved in acute intoxication was heroin (Table S3).

### 3.8 | Suicide: Simple and complex suicide

During the analyses of the circumstantial data of the cases under investigation, we observed that in 13 cases the causes of death was registered as suicide, with 9 cases in 2018 and 4 in 2019. Simple suicides are events put in place with a single modality of death, while complex suicides are characterized by more than one modality.

The individuals that committed suicide were nine males and seven females with a most represented range‐of‐age 41–50 (5 cases). According to literature, suicide is usually associated to psychiatric disorders, drug abuse, psychological conditions or previous suicide attempts [19]. Indeed, we noticed that the subjects of our study that committed suicides were suffering from major depressive disorder, had previously attempted suicide, had psychiatric diseases (as borderline disorders, schizophrenia, bulimia), underwent mandatory medical treatments or were suffering from substances dependance. Only two individuals did not present any of these conditions: a male belonging to the range‐of‐age 21–30 (case no. 17) that hung himself, resulted to be negative to toxicological analyses, and never attempted suicide before and a female (age: 21–30) that fell from height with positive findings to fluoxetine.

Moreover, from our data, we can differentiate suicide cases according to the manner of death: simple suicides (12 cases) and complex suicides (5 cases), as reported in Table S3.

In five cases (three males and two females), individuals decided to end their life by falling from height. Their corpses were discovered nearby the houses, and all of them were under the influence of some substance (e.g., antidepressants, benzodiazepines and stimulants, or drugs of abuse).

In three cases, individuals accomplished suicide by acute intoxication of different substances: delorazepam and alprazolam; tramadol; cyanide. Additionally, in these cases the deceased were suffering from major depressive disorder.

Delorazepam, alprazolam and tramadol were regularly quantitated (see Tables 1 and 2), while cyanide was only evaluated under a qualitative point of view. Since testing tubes containing potassium cyanide were discovered on the suicide scene, the Judicial Authority requested a mere confirmation of the presence of the substance in the biological matrices sampled from the corpse. Quantitation data are therefore not available (Tables 1 and 2).

Five individuals committed suicide by combination of acute intoxication and other suicidal means (complex suicide section of Table S3). They were four males and one female, with circumstantial data that mentioned drug addiction, depression, psychosis, and schizophrenia. Two individuals died of cocaine or delorazepam and bromazepam acute intoxication combined with gas inhalation; both corpses were discovered in the car. Three of them died of butane or cocaine or a mix of prescription drugs acute intoxication combined with suffocation (mix of drugs: clozapine, alprazolam, diazepam, paroxetine, olanzapine, pregabalin, clonazepam, lorazepam and delorazepam); in one case PBS was involved, whereas in another case the individual died from hanging (combined with cocaine). As for the last case, the manner of suffocation was not described in circumstantial data.

Finally, two individuals killed themself without being under the influence of any substance and two subjects without suffering from mental illness or having an abuse of substances history (Table S3).

### 3.9 | Homicide

The analyses of the circumstantial data and autopsy reports revealed homicide as cause of death in four individuals. All the subjects included in this category were male with a range of age 41–50 in two cases and 51–60 and 61–70 in the other two cases. Two cases were negative to toxicological analyses and died after being hit by a car (1 case) and after more than 11 shotguns to the chest (1 case). The other two individuals died as a consequence of beatings (case no. 42) and after being pushed down from the 6th floor (case no. 81). These last two cases were positive to toxicological analyses, precisely to the analyses of BAC: 4.35 g/L was the concentration of alcohol detected in case no. 81 and 2.56 g/L was the value detected in case no. 42. The effects of alcohol on the human body increases with the increasing of the BAC: from 2.0 to 3.0 g/L the effects on an occasional drinker are aggressiveness, verbal incoherence, lethargy and vomit; whereas from 3.0 to 4.0 g/L, the effects are nausea and coma [15] (Table S3).

### 3.10 | Natural death: Heart failure

The toxicological analyses performed on deceased individuals for which the cause of death was ascribable to heart failure were positive in 58.0% of cases (30 out of 52 individuals) and negative in 22 cases (42.0%). From the total number of cases belonging to this category, 11 individuals were females without a characteristic range‐of‐age, whereas 41 subjects were male with two major range‐of‐age: 41–50 and 31–40. In these cases, the toxicological analyses were performed for completeness of the medico‐legal investigations, providing further information on the hours preceding the death of the subjects (Table S3).

## 4 | DISCUSSION

### 4.1 | Drug related and non‐drug related deaths

After a careful analysis of the results, the concentrations obtained in drug‐related deaths (accidental death by acute intoxication, simple and complex suicide) and in non‐drug related deaths (car accident, simple and complex suicide, and heart failure) were compared (Table 1). Drug‐related deaths by a single substance were represented in 26 cases of acute intoxication and two cases of suicide (one complex and one simple suicide), for a total of 28 individuals of fatal acute intoxication. The illicit drug responsible for most cases of acute intoxication (16 cases ‐ 29.6%) (one of them was a complex suicide) was cocaine, followed by morphine (4 cases – 7.4%) and methadone (3 cases – 5.4%) (Table 1). These data are partially in contrast with another study [6], that noted the same most representative molecules in acute intoxication deaths but with morphine as the substance most detected (25.8%), followed by cocaine (9.5%) and methadone (7.7%). Also, considering the population of New York, opiates (heroin), cocaine and alcohol were the major substances detected in accidental fatal acute intoxications both together and as single drug administration [20]; whereas in opposite to our results, the most common drugs detected in 161 cases of fatal acute intoxication from 2015 to 2019 in Cairo (Egypt) were opioids [21]. Moreover, considering the events in which fatal acute intoxication was due to more than one substance, we obtain a total number of 54 cases. According to our data, 4.0% of all the autopsies taken in consideration (54 out of 1323) during a 2‐year period, resulted to be acute intoxication. Considering this result, it seems that in Finland the double of the population died for acute intoxication between 2000 and 2017 (8.0% of the population died for acute intoxication with gender percentages superimposable to our study) [22]. Assuming that, all the autopsies for which a toxicological evaluation was not requested could be considered as effectively negative, we feel confident in stating that Italy has a lower percentage of fatal intoxication if compared to other scenarios, as the Finnish one [22].

Whereas, in Sweden during a 10‐year period, fatal acute intoxication (both accidental and suicide) was ascribable to a single substance in 22.0% of the cases (in contrast with our study, where 51.8%, 28 out of 54 acute intoxications of suicides or accidental acute intoxication were caused by a single substance, Table 1). While, considering only the cases in which toxicological analyses were assessed, death by acute intoxication was 39.7% (54 out of 136).

Seven cases belonged to the group of non‐drug related deaths with a single substance: one simple suicide, two complex suicides, three natural deaths caused by heart failure and one car accident. The most common drug detected was cocaine ranging from 0.33 to 19.8 μg/ml in cardiac blood (average of 5.37 μg/ml) and from 0.49 to 18.78 μg/ml in femoral blood (average of 5.28 μg/ml) in cases of drug‐related deaths, while in non‐drug related death the single case detected reported 0.05 μg/ml in cardiac blood and 0.14 μg/ml in femoral blood, represented by one case. The concentration of cocaine obtained in drug‐related deaths can be considered lethal as reported in literature [23]. However, even if some comparison between drug related deaths and non‐drug related deaths cannot be performed, all the concentrations obtained are in accordance with literature concentrations [23, 24].

A comparison between acute intoxication suicides and accidental suicides by single drug might be considered as complicated: the only one drug that was present in both categories was cocaine. This molecule was detected in one case of complex suicide, in which this illicit substance was the main cause of death, with 1.53 μg/ml in cardiac blood and 0.1 μg/ml in femoral blood and in 15 cases of accidental acute intoxication death (cardiac blood concentration: 5.37 μg/ml, average calculated on 13 cases, considering that in two individuals, nos. 35 and 61, the cardiac blood was missing; femoral blood concentration: 5.28 μg/ml, average calculated on 14 cases, because in one case, no. 35, the femoral blood was missing).

### 4.2 | Most representative profiles extrapolated from this study

In a 2‐year period, 136 toxicological analyses were performed on deceased that were carried to the Bureau of Legal Medicine of the University of Milan. From those cases a study, based on data collection, has been done investigating the clinical records, the cause and the manner of death and the results of the toxicological analyses of each case.

From this records study it was possible to identify the most representative profiles of individuals who were transported to the Bureau of Legal Medicine of the University of Milan for autopsy investigations. A Caucasian and Italian male, with a range‐of‐age of 41–50 (mean 45.95 ± 2.6), that died for acute intoxication of cocaine. Considering the suicide causes of death, the sex and the age are equally represented; therefore, the typical individual could be a Caucasian and Italian male or female with a range‐of‐age that goes from 31 to 50 years died for simple suicide caused by acute intoxication or a complex suicide caused by acute intoxication and suffocation. From the analyses of killed individuals, we can identify a Caucasian and Italian male with a range‐of‐age 41–50 (mean value: 50.25 ± 10.6).

The most representative individual that died for accidental death is a male with a range‐of‐age 21–40 (mean 31.75 ± 6.4) that died in a car accident without any toxicological evidence.

And finally, the toxicological analyses were requested for 136 cases, 101 (74.3%) of them were positive to toxicological investigations and in 55 (40.4% in respect with 136 individuals; 54.5% in relation to the positive results) the toxicological data provided to the forensic practitioner were essential to determine the cause and manner of death of the individuals.

In 46 cases, the results were essential to determine the cause of death of the individuals, detecting the substances responsible of involuntary acute intoxication, whereas three individuals committed simple suicide with acute intoxication by prescription drugs (case nos. 11 and 82) and by cyanide in case no.109. In cases in which the cause of death was due to complex suicide (five individuals in total), the toxicological analyses were essential to determine the acute intoxication by substances combined with different means used to commit suicide. In one case (no.110) the accidental death by fall from height of a man with range‐of‐age 11–20, the detection of LSD contributed to determine the unvoluntary fall of the individual. Indeed, LSD concentrations detected in biological samples are suitable to cause an altered psychomotor state, represented by visual and sensory alterations sufficient to determine a distorted perception of the reality. Considering, relatives' interviews, circumstantial data, medico‐legal findings and toxicological results, the forensic practitioner decided for a probable accidental fall from height. Furthermore, in 46 cases, toxicological analyses were essential to complete, clarify and add important information to the medico‐legal investigations about the last hours of people's lives, making the analytical data an important support for the medico‐legal practitioner during forensic investigations. Negative toxicological results obtained from the remaining 35 cases were important in the forensic investigations underlining the absence of drugs, substances of abuse and alcohol in the hours preceding the death of a subject.

## 5 | CONCLUSION

Thanks to this study we have outlined the typical subject that were carried at the Bureau of Legal Medicine of Milan for autopsy investigation, representative for a large metropolitan area such as the Milanese one: a Caucasian Italian male, with a range‐of‐age of 41–50 (mean 45.95 ± 2.6), that died of cocaine acute intoxication. Although the period investigated is restricted to 2 years, this study lays the groundwork for future investigations on longer periods and larger populations. Moreover, it was noted that the integration with toxicological data resulted, in 39.7% of the cases requested by the Judicial Authority, essential in forensic investigations and highlighted how this type of investigation should be considered fundamental in finding the cause and manner of death of an individual.

## ETHICS STATEMENT

This article does not contain any studies with animals performed by any of the authors. All procedures performed in studies involving human participants were in accordance with the ethical standards of the institutional and/or national research committee and with the 1964 Helsinki declaration and its later amendments or comparable ethical standards.

## DISCLOSURE

All the data toxicological analyses were collected for other purposes, and they were enrolled in our study with blinded information; therefore, the data cannot be linked to the identity of the individuals.

## Supporting information


Table S1
Table S2
Table S3
