# Supplementary material for: Postmortem forensic toxicology cases: A retrospective review from Milan, Italy
Source: J Forensic Sci. 2022 May 4;67(4):1640–50. doi: 10.1111/1556-4029.15050 (PMC9325463; doi:10.1111/1556-4029.15050)
Supplement: Supplementary file 1 — Table S1 Table S2 Table S3 [file JFO-67-1640-s001.docx]

**Supplementary Material**

TABLE S1 Table resuming the demographic data of each case, the setting of body discovery, the anamnesis of individuals and the biological samples analyzed for the toxicological analyses

| **N.** | **Sex** | **Age** | **Range of age** | **Ethnicity** | **Setting of body discovery** | **Anamnesis** | **Biological samples** |
| --- | --- | --- | --- | --- | --- | --- | --- |
| **1** | M | 32 | 31-40 | Caucasian | / | Chronic heart disease | Peripheral blood  Cardiac blood  Urine  Gastric content |
| **2** | M | 60 | 51-60 | Caucasian | / | Car accident | Peripheral blood  Cardiac blood  Liver  Gastric content |
| **3** | F | 39 | 31-40 | Caucasian | House | Depression | Peripheral blood  Cardiac blood |
| **4** | M | 46 | 41-50 | Caucasian | / | / | Peripheral blood  Cardiac blood  Urine  Gastric content |
| **5** | M | 53 | 51-60 | Caucasian | House | Drugs rehabilitation for heroin addiction  Withdrawal symptoms | Peripheral blood  Cardiac blood  Urine  Gastric content |
| **6** | M | 34 | 31-40 | Caucasian | / | / | Peripheral blood  Urine  Brain |
| **7** | M | 23 | 21-30 | Caucasian | / | Car accident | Peripheral blood  Cardiac blood  Urine  Gastric content  Bile |
| **8** | F | 43 | 41-50 | Caucasian | / | Suspected acute narcotism by drugs of abuse | Peripheral blood  Cardiac blood  Urine  Gastric content |
| **9** | M | 42 | 41-50 | Caucasian | Airport | / | Peripheral blood  Cardiac blood  Urine  Gastric content |
| **10** | M | 39 | 31-40 | Caucasian | / | Maybe use of cocaine and hashish in the days before death | Peripheral blood  Brain  Urine |
| **11** | F | 39 | 31-40 | Caucasian | House | Depression  Suicide with empty bottle of alprazolam  Previous suicide attempts | Peripheral blood  Cardiac blood  Urine  Gastric content  Brain |
| **12** | M | 40 | 31-40 | Caucasian | House | Fallen from the ninth floor: suicide  Psychiatric pathologies | Peripheral blood  Cardiac blood  Liver  Gastric content |
| **13** | F | / | / | Caucasian | / | / | Cardiac blood  Urine |
| **14** | M | 85 | 81-90 | Caucasian | Hotel | Diazepam on the bedside table and bottle of alcohol | Peripheral blood  Cardiac blood  Urine  Gastric content |
| **15** | M | 53 | 51-60 | Caucasian | Park | / | Peripheral blood  Cardiac blood  Urine  Gastric content |
| **16** | M | 52 | 51-60 | Caucasian | / | Previous drug addiction and alcoholism | Peripheral blood  Cardiac blood  Urine  Gastric content |
| **17** | M | 23 | 21-30 | Caucasian | House | Hanged in the house  No previous suicide attempts | Peripheral blood  Cardiac blood  Urine  Gastric content |
| **18** | M | 46 | 41-50 | Caucasian | Car | Drug addicted, suicide with farewell letter  Found the body in a car with a tank of GPL | Peripheral blood  Cardiac blood  Liver  Gastric content  Bile |
| **19** | M | / | / | Caucasian | / | / | Peripheral blood  Cardiac blood  Urine  Gastric content |
| **20** | M | 44 | 41-50 | Caucasian | / | Suicide with plastic bag  psychiatric treatment (schizophrenia) | Peripheral blood  Cardiac blood  Urine  Gastric content  Liver  Brain  Lung  Fat |
| **21** | M | 25 | 21-30 | Caucasian | / | / | Peripheral blood  Cardiac blood  Urine  Gastric content |
| **22** | M | 51 | 51-60 | Caucasian | House | Found hanged in the courtyard of the house  Ex drug addicted treated in the community about 10 years ago | Peripheral blood  Cardiac blood  Liver  Gastric content  Urine  Bile |
| **23** | M | 55 | 51-60 | Caucasian | / | / | Brain  Peripheral blood  Urine  Gastric content |
| **24** | M | 29 | 21-30 | Caucasian | / | Car accident | Peripheral blood  Cardiac blood  Urine  Gastric content |
| **25** | M | 32 | 31-40 | Caucasian | / | / | Brain |
| **26** | F | 23 | 21-30 | Caucasian | Park | / | Peripheral blood  Cardiac blood  Urine  Bile |
| **27** | F | 48 | 41-50 | Caucasian | / | gunshot to the head, anxiety spectrum disorder in therapy by a psychologist | Peripheral blood  Cardiac blood  Urine  Gastric content |
| **28** | M | 63 | 61-70 | Caucasian | House | found in a house, in a car with at least 11 gunshots to the chest | Peripheral blood  Cardiac blood  Urine  Gastric content |
| **29** | M | 48 | 41-50 | Caucasian | House | Found on the living room floor, supine  Major depressive disorder, anxiety disorder | Peripheral blood  Cardiac blood  Urine  Gastric content |
| **30** | M | 47 | 41-50 | Caucasian | / | / | Peripheral blood  Cardiac blood  Urine  Gastric content |
| **31** | M | 48 | 41-50 | Caucasian | / | Cold weapon  Suicide | Peripheral blood  Cardiac blood  Urine  Gastric content |
| **32** | M | 39 | 31-40 | Caucasian | / | Car accident | Peripheral blood  Cardiac blood  Urine  Gastric content |
| **33** | M | 24 | 21-30 | Caucasian | / | / | Peripheral blood  Cardiac blood  Urine  Gastric content |
| **34** | M | 25 | 21-30 | Caucasian | / | / | Peripheral blood  Urine  Gastric content |
| **35** | M | 51 | 51-60 | Caucasian | Car | Body was found in a car with head hanging in the passenger seat | Liver  Brain  Bile |
| **36** | M | 26 | 21-30 | Caucasian | House | Found on the bed by his father  Heart problems with abnormal electrocardiogram  Closed to the body discovered close envelope of cocaine | Peripheral blood  Cardiac blood  Urine  Bile  Gastric content |
| **37** | M | 45 | 41-50 | Caucasian | House | Corpse found supine on the sofa in the living room  Depression | Peripheral blood  Cardiac blood  Urine  Gastric content  Liver |
| **38** | M | 64 | 61-70 | Caucasian | Jail | / | Peripheral blood  Cardiac blood  Urine  Gastric content |
| **39** | M | 25 | 21-30 | Caucasian | / | Accident at work | Peripheral blood  Cardiac blood  Urine  Gastric content |
| **40** | M | 84 | 81-90 | Caucasian | Car | Depression  Found inside the car, with a tool to convey the exhaust gases inside the car | Peripheral blood  Cardiac blood  Urine  Gastric content |
| **41** | M | 55 | 51-60 | Caucasian | / | Car accident | Brain  Cardiac blood  Gastric content |
| **42** | M | 41 | 41-50 | Caucasian | / | Beatings | Peripheral blood  Cardiac blood  Urine  Gastric content |
| **43** | M | 13 | 11-20 | Caucasian | / | / | Peripheral blood  Cardiac blood  Urine  Gastric content |
| **44** | F | 35 | 31-40 | Caucasian | / | / | Peripheral blood  Cardiac blood  Liver  Gastric content  Brain  Psoas  Kidney  Liquor  Lung |
| **45** | M | 67 | 61-70 | Caucasian | Hospital | / | Peripheral blood  Cardiac blood  Liver  Kidney |
| **46** | F | 73 | 71-80 | Caucasian | / | Precipitated from the 3^rd^ floor. A  Already attempted suicide with benzodiazepines | Peripheral blood  Cardiac blood  Urine  Gastric content |
| **47** | M | / | / | Caucasian | / | / | Peripheral blood  Cardiac blood  Urine  Gastric content |
| **48** | M | 66 | 61-70 | Caucasian | Hospital | Hypertension and heart disease | Peripheral blood  Cardiac blood  Brain  Gastric content |
| **49** | F | 38 | 31-40 | Caucasian | / | Car accident | Peripheral blood  Cardiac blood  Urine  Gastric content |
| **50** | M | 33 | 31-40 | Caucasian | / | Psychiatric disease | Peripheral blood  Cardiac blood  Urine  Gastric content |
| **51** | M | 29 | 21-30 | Caucasian | / | Car accident | Peripheral blood  Cardiac blood  Urine  Gastric content |
| **52** | M | 47 | 41-50 | Caucasian | / | Attended drug rehabilitation community | Peripheral blood  Cardiac blood  Urine  Gastric content |
| **53** | F | 80 | 71-80 | Caucasian | / | Hypertension, depression | Peripheral blood  Cardiac blood  Urine  Gastric content |
| **54** | M | 24 | 21-30 | Caucasian | / | Car accident | Peripheral blood  Cardiac blood  Urine  Gastric content |
| **55** | F | 27 | 21-30 | Caucasian | / | Likely drug addict  Found with a water pipe beside it  In 2014 start of heroin use  Use of cocaine | Peripheral blood  Cardiac blood  Urine  Gastric content |
| **56** | F | 49 | 41-50 | Caucasian | House | Supine corpse on the sofa bed  Dish containing heroin  Psychiatric pathology  Previous involuntary medical treatment | Peripheral blood  Gallbladder  Liver  Spleen  Kidney  Gastric content |
| **57** | M | 69 | 61-70 | Caucasian | / | Car accident | Peripheral blood  Cardiac blood  Urine  Gastric content |
| **58** | M | 42 | 41-50 | Caucasian | / | Depression in cure with a psychiatrist  Last suicide attempt in 2018 | Peripheral blood  Cardiac blood  Urine  Gastric content |
| **59** | M | 69 | 61-70 | Caucasian | / | Car accident | Peripheral blood  Cardiac blood  Urine  Gastric content |
| **60** | M | 23 | 21-30 | Latin American | / | / | Peripheral blood  Cardiac blood  Urine  Gastric content |
| **61** | M | 33 | 31-40 | Caucasian | House | Voluntary precipitation from the 4^th^ floor | Peripheral blood  Urine  Liver  Gastric content |
| **62** | M | 39 | 31-40 | Caucasian | / | Homeless | Peripheral blood  Cardiac blood  Urine  Gastric content |
| **63** | M | 51 | 51-60 | Caucasian | / | **/** | Peripheral blood |
| **64** | F | 27 | 21-30 | / | House | Discovered a corpse in bed with syringes next to it  Drugs of abuse in the bag | Peripheral blood  Cardiac blood  Urine  Gastric content |
| **65** | M | 35 | 31-40 | Caucasian | Car | **/** | Urine  Gastric content  Spleen exudate  Kidney  Brain  Liver |
| **66** | M | 41 | 41-50 | Caucasian | / | drug addict, in the past followed by communities for psychiatric and drug addiction disorders. psychotropic drugs, paranoid schizophrenia | Peripheral blood  Urine  Liver  Gastric content |
| **67** | M | 38 | 31-40 | Caucasian | / | mitral valve disease, not in therapy  Hypertension  Heart disease | Peripheral blood  Cardiac blood  Gastric content  Bile  Brain  Kidney  Spleen |
| **68** | M | 5 months | > 1 year | Caucasian | / | / | Peripheral blood  Cardiac blood  Urine  Gastric content |
| **69** | M | 44 | 41-50 | Caucasian | / | / | Peripheral blood  Cardiac blood  Liver  Gastric content |
| **70** | M | 46 | 41-50 | Caucasian | / | Use of cocaine | Peripheral blood  Cardiac blood  Bile |
| **71** | M | 34 | 31-40 | Caucasian | / | Car accident | Peripheral blood |
| **72** | F | 21 | 21-30 | Caucasian | / | / | Peripheral blood  Cardiac blood  Urine  Gastric content  Liver |
| **73** | F | 71 | 71-80 | Caucasian | / | / | Peripheral blood  Cardiac blood  Urine  Gastric content  Liver |
| **74** | M | 38 | 31-40 | Caucasian | / | Car accident | Peripheral blood  Cardiac blood  Urine  Gastric content |
| **75** | M | 45 | 41-50 | Caucasian | House | **/** | Peripheral blood  Cardiac blood  Urine  Gastric content |
| **76** | M | 59 | 51-60 | Caucasian | / | / | Peripheral blood  Cardiac blood  Urine  Gastric content |
| **77** | M | 22 | 21-30 | Caucasian | / | / | Brain  Kidney  Liver  Psoas  Hair  Pubic hair  Larvae |
| **78** | M | 46 | 41-50 | Caucasian | / | / | Peripheral blood  Cardiac blood  Urine  Gastric content |
| **79** | F | 77 | 71-80 | Caucasian | / | / | Peripheral blood  Cardiac blood  Urine  Gastric content  Hair |
| **80** | M | 44 | 41-50 | Caucasian | / | / | Peripheral blood  Cardiac blood  Urine  Gastric content |
| **81** | M | 42 | 41-50 | Caucasian | / | Push down by the 6^th^ floor | Peripheral blood  Cardiac blood  Urine  Gastric content |
| **82** | F | 47 | 41-50 | Caucasian | / | Previous suicide attempts | Peripheral blood  Cardiac blood  Urine  Gastric content |
| **83** | F | 65 | 61-70 | Caucasian | Hotel | Found in the hotel  Accidental death by substances  Financial distress in recent times | Peripheral blood  Cardiac blood  Urine  Gastric content |
| **84** | F | 44 | 41-50 | Caucasian | / | Suicide attempts in Jan and Feb 2018 | Peripheral blood  Cardiac blood  Urine  Gastric content |
| **85** | F | 3 months | > 1 year | Caucasian | / | / | Peripheral blood  Cardiac blood  Urine  Gastric content |
| **86** | M | 30 | 21-30 | Caucasian | House | Fall from the 6^th^ floor  Depressed followed by a psychiatris | Peripheral blood  Cardiac blood  Urine  Gastric content  Hair1/2 cm  Hair matrices 1 cm |
| **87** | F | / | / | Caucasian | / | / | Peripheral blood  Cardiac blood  Urine  Gastric content |
| **88** | M | 47 | 41-50 | Caucasian | / | Depression | Peripheral blood  Cardiac blood  Urine  Gastric content |
| **89** | M | 36 | 31-40 | Caucasian | / | psychiatric and drug addict patients. Diagnosis of personality disorder in 2016 | Peripheral blood  Cardiac blood  Urine  Gastric content |
| **90** | M | 66 | 61-70 | Caucasian | / | Heart bypass 6 months earlier | Peripheral blood  Cardiac blood  Urine  Gastric content |
| **91** | M | 45 | 41-50 | Caucasian | House | Previous drug addiction (cocaine) | Peripheral blood  Cardiac blood  Urine  Gastric content |
| **92** | M | 42 | 41-50 | Caucasian | / | Cardiomyopathy | Peripheral blood  Cardiac blood  Urine  Gastric content |
| **93** | M | / | / | Caucasian | / | Infanticide | Hair 4 cm prox  Hair 4 cm inter  Hair 4 cm distali  Bile  Blood |
| **94** | M | 84 | 81-90 | Caucasian | / | / | Peripheral blood  Cardiac blood  Urine  Gastric content |
| **95** | F | 31 | 31-40 | Caucasian | House | Previous days strongly depressed moods with suicidal intentions  Under Treatment at the Psycho-social centre  Suicide in the bathroom by plastic bag suffocation | Peripheral blood  Cardiac blood  Urine  Gastric content |
| **96** | F | 42 | 41-50 | Caucasian | / | / | Peripheral blood  Cardiac blood  Urine  Gastric content |
| **97** | M | 47 | 41-50 | Caucasian | / | / | Peripheral blood  Cardiac blood  Liver  Gastric content |
| **98** | M | 39 | 31-40 | Caucasian | / | Drug addict for 5/7 years  Unvoluntary medical treatment in the previous year | Peripheral blood  Cardiac blood  Urine  Gastric content |
| **99** | M | 25 | 21-30 | Caucasian | / | Alcohol and drug abuse | Peripheral blood  Cardiac blood  Urine  Gastric content  Nasal swabs |
| **100** | M | 35 | 31-40 | Caucasian | / | / | Peripheral blood  Cardiac blood  Urine  Gastric content |
| **101** | F | 54 | 51-60 | Asiatic | House | Accidental fall | Peripheral blood  Cardiac blood  Urine  Gastric content |
| **102** | F | 54 | 51-60 | Caucasian | House | Strangulation with belt, found the body at home | Peripheral blood  Urine  Liver  Gastric content |
| **103** | F | 20 | 11-20 | Caucasian | House | precipitation from the 7^th^ floor  Borderline Personality Disorder  Depression.  Previous suicide attempts  Farewell letter | Brain  Peripheral blood  Cardiac blood  Urine  Gastric content |
| **104** | M | 67 | 61-70 | Caucasian | / | / | Peripheral blood |
| **105** | F | 56 | 51-60 | Caucasian | House | Found in the room, semi-lying on the ground | Peripheral blood  Cardiac blood  Urine  Gastric content |
| **106** | F | 84 | 81-90 | Caucasian | House | Found a corpse inside his home | Peripheral blood  Cardiac blood  Liver |
| **107** | M | 55 | 51-60 | Caucasian | / | Hit by a car | Peripheral blood  Brain |
| **108** | M | / | / | Caucasian | / | / | Peripheral blood  Liver  Brain  Urine  Gastric content  Pubic hair |
| **109** | M | 57 | 51-60 | Caucasian | Work | Suicide with cyanide  Farewell letter | Peripheral blood |
| **110** | M | 19 | 11-20 | Caucasian | House | Accidental fall | Peripheral blood  Cardiac blood  Urine  Gastric content |
| **111** | M | 21 | 21-30 | Caucasian | House | Found dead in the house, prone with his face on the pillow | Peripheral blood  Cardiac blood  Urine  Gastric content |
| **112** | M | 33 | 31-40 | Caucasian | Train | Found on the train, probable illness | Peripheral blood  Cardiac blood  Urine  Gastric content |
| **113** | M | 48 | 41-50 | Caucasian | House | Found inside his home  Previous use of cocaine | Peripheral blood  Cardiac blood  Urine  Gastric content |
| **114** | M | 28 | 21-30 | Caucasian | Swimming pool | / | Peripheral blood  Cardiac blood  Urine  Gastric content |
| **115** | M | 60 | 51-60 | Caucasian | / | / | Peripheral blood  Cardiac blood  Urine  Gastric content |
| **116** | M | 43 | 41-50 | Caucasian | / | / | Peripheral blood  Cardiac blood  Liver  Gastric content |
| **117** | F | 27 | 21-30 | Caucasian | / | Drug addiction  Likely acute intoxication | Peripheral blood  Cardiac blood  Liver  Gastric content  Bile  Nasal swabs |
| **118** | M | 30 | 21-30 | Caucasian | Park | / | Peripheral blood  Cardiac blood  Urine  Gastric content |
| **119** | F | 57 | 51-60 | Caucasian | House | **/** | Peripheral blood  Cardiac blood  Urine  Gastric content |
| **120** | M | 82 | 81-90 | Caucasian | / | Accidental death: fall | Peripheral blood  Cardiac blood  Urine  Gastric content |
| **121** | M | 54 | 51-60 | Caucasian | House | found on the sofa in his home  Depression  Alcohol and drug abuse | Peripheral blood  Cardiac blood  Urine  Gastric content |
| **122** | M | / | / | Caucasian | / | / | Brain  Liver  Spleen  Urine  Gastric content |
| **123** | M | 57 | 51-60 | Caucasian | House | Drug addiction | Peripheral blood  Cardiac blood  Urine  Gastric content |
| **124** | M | 47 | 41-50 | Caucasian | Hospital | Found on the sidewalk next to the car | Peripheral blood  Cardiac blood  Urine  Gastric content |
| **125** | F | 43 | 41-50 | Latin American | / | / | Peripheral blood  Cardiac blood  Urine  Gastric content |
| **126** | F | 82 | 81-90 | Caucasian | / | Depression for 20 years | Peripheral blood  Cardiac blood  Urine  Gastric content |
| **127** | M | 24 | 21-30 | Caucasian | House | Accidental fall | Peripheral blood  Cardiac blood  Urine  Gastric content |
| **128** | M | 45 | 41-50 | Caucasian | Street | / | Peripheral blood  Cardiac blood  Urine  Gastric content |
| **129** | M | 33 | 31-40 | Caucasian | Park | Previous acute narcotism by heroin | Peripheral blood  Urine  Liver  Gastric content  Bile |
| **130** | M | 20 | 11-20 | Caucasian | Hotel | Found in a hotel room supine on the bed | Peripheral blood  Cardiac blood  Urine  Bile |
| **131** | M | 63 | 61-70 | Caucasian | Hospital | / | Peripheral blood  Cardiac blood  Liver  Urine |
| **132** | M | 46 | 41-50 | Caucasian | / | Car accident | Peripheral blood  Cardiac blood  Urine  Gastric content |
| **133** | M | 52 | 51-60 | Asiatic | / | / | Peripheral blood  Cardiac blood  Urine  Gastric content |
| **134** | F | 21 | 21-30 | Black | House | Precipitation | Peripheral blood  Urine  Liver  Kidney  Spleen  Gastric content |
| **135** | M | 30 | 21-30 | Caucasian | Jail | / | Peripheral blood  Cardiac blood  Urine  Gastric content |
| **136** | M | 46 | 41-50 | Caucasian | / | Suspected drug addiction  Previous depression, treatment with a psychologist | Peripheral blood  Cardiac blood  Urine  Gastric content |

TABLE S2 The table summarizes drugs and illicit substances detected via toxicological analysis. For each substance, we are reporting the total of positive cases, the percentage compared to total cases considered (136), the percentage on positive results (101), limit of detection (LOD) and lowest limit of quantification (LLOQ)

| Drug | N° of times the molecules were detected one time in the cases under investigation | Percentage of drugs on total n° of cases (136) | Percentage of drugs on positive cases (101) | Limit of detection (LOD) | Lowest limit of quantification  (LLOQ) |
| --- | --- | --- | --- | --- | --- |
| Benzoylecgonine | 38 | 27.9% | 37.6% | 3 ng/mL | 30 ng/mL |
| Cocaine | 37 | 27.2% | 36.6% | 2 ng/mL | 20 ng/mL |
| Ethanol | 36 | 26.5% | 35.6% | 0.001 g/L | 0.01 g/L |
| Diazepam | 22 | 16.1% | 21.8% | 5 ng/mL | 50 ng/mL |
| Free morphine | 13 | 9.5% | 12.8% | 0.5 ng/mL | 5 ng/mL |
| Delorazepam | 9 | 6.6% | 8.9% | 5 ng/mL | 55 ng/mL |
| Zolpidem | 8 | 5.8% | 7.9% | 4 ng/mL | 50 ng/mL |
| Alprazolam | 7 | 5.1% | 6.9% | 0.5 ng/mL | 5 ng/mL |
| Bromazepam | 7 | 5.1% | 6.9% | 2 ng/mL | 20 ng/mL |
| Methadone | 6 | 4.3% | 5.8% | 5 ng/mL | 50 ng/mL |
| THC | 6 | 4.3% | 5.8% | 0.1 ng/mL | 4 ng/mL |
| Cotinine | 4 | 2.9% | 3.9% | 0.1 ng/mL | 1 ng/mL |
| Ketamine | 4 | 2.9% | 3.9% | 10 ng/mL | 110 ng/mL |
| Lormetazepam | 4 | 2.9% | 3.9% | 0.4 ng/mL | 5 ng/mL |
| Midazolam | 4 | 2.9% | 3.9% | 2 ng/mL | 20 ng/mL |
| Paroxetine | 4 | 2.9% | 3.9% | 0.1 ng/mL | 1 ng/mL |
| Tramadol | 4 | 2.9% | 3.9% | 5 ng/mL | 60 ng/mL |
| Amlodipine | 3 | 2.2% | 2.9% | 0.5 ng/mL | 5 ng/mL |
| Codeine | 3 | 2.2% | 2.9% | 1 ng/mL | 15 ng/mL |
| EDDP | 3 | 2.2% | 2.9% | 1 ng/mL | 10 ng/mL |
| Fluoxetine | 3 | 2.2% | 2.9% | 10 ng/mL | 120 ng/mL |
| MDA | 3 | 2.2% | 2.9% | 4 ng/mL | 50 ng/mL |
| Nordiazepam | 3 | 2.2% | 2.9% | 1 ng/mL | 15 ng/mL |
| Quetiapine | 3 | 2.2% | 2.9% | 10 ng/mL | 150 ng/mL |
| THCCOOH | 3 | 2.2% | 2.9% | 0.1 ng/mL | 1 ng/mL |
| Venlafaxine | 3 | 2.2% | 2.9% | 10 ng/mL | 150 ng/mL |
| Heroin | 2 | 1.5% | 1.9% | 0.1 ng/mL | 1 ng/mL |
| Phenobarbital | 2 | 1.5% | 1.9% | 100 ng/mL | 2 μg/mL |
| Fentanyl | 2 | 1.5% | 1.9% | 0.1 ng/mL | 2 ng/mL |
| MDMA | 2 | 1.5% | 1.9% | 4 ng/mL | 50 ng/mL |
| Methamphetamine | 2 | 1.5% | 1.9% | 0.5 ng/mL | 5 ng/mL |
| Oxycodone | 2 | 1.5% | 1.9% | 0.8 ng/mL | 10 ng/mL |
| Sertraline | 2 | 1.5% | 1.9% | 4 ng/mL | 45 ng/mL |
| Haloperidol | 1 | 0.73% | 0.99% | 0.1 ng/mL | 2 ng/mL |
| Allopurinol | 1 | 0.73% | 0.99% | 150 ng/mL | 1.5 μg/mL |
| Butane | 1 | 0.73% | 0.99% | 10 ng/mL | 100 ng/mL |
| 6-MAM | 1 | 0.73% | 0.99% | 0.3 ng/mL | 4 ng/mL |
| Bisoprolol | 1 | 0.73% | 0.99% | 700 ng/mL | 9 ng/mL |
| Bupropion | 1 | 0.73% | 0.99% | 500 ng/mL | 7 μg/mL |
| Ceftriaxone | 1 | 0.73% | 0.99% | 900 ng/mL | 10 μg/mL |
| Ceftazidime | 1 | 0.73% | 0.99% | 1 μg/mL | 12 μg/mL |
| Cyanide | 1 | 0.73% | 0.99% | / | / |
| Ecgonine methyl ester | 1 | 0.73% | 0.99% | 1 ng/mL | 10 ng/mL |
| Flurazepam | 1 | 0.73% | 0.99% | 1 ng/mL | 15 ng/mL |
| Hydrochlorothiazide | 1 | 0.73% | 0.99% | 4 ng/mL | 5 ng/mL |
| Irbesartan | 1 | 0.73% | 0.99% | 90 ng/mL | 1 μg/mL |
| Ketorolac | 1 | 0.73% | 0.99% | 40 ng/mL | 450 ng/mL |
| Lorazepam | 1 | 0.73% | 0.99% | 6 ng/mL | 70 ng/mL |
| LSD | 1 | 0.73% | 0.99% | 0.03 ng/mL | 0.4 ng/mL |
| MDPHP | 1 | 0.73% | 0.99% | 9 ng/mL | 100 ng/mL |
| MDPV | 1 | 0.73% | 0.99% | 10 ng/mL | 100 ng/mL |
| Methomyl | 1 | 0.73% | 0.99% | 100 ng/mL | 1 μg /mL |
| Mirtazapine | 1 | 0.73% | 0.99% | 25 ng/mL | 250 ng/mL |
| Naloxone | 1 | 0.73% | 0.99% | 0.5 ng/mL | 5 ng/mL |
| Nicotine | 1 | 0.73% | 0.99% | 0.4 ng/mL | 4 ng/mL |
| Olanzapine | 1 | 0.73% | 0.99% | 1 ng/mL | 10 ng/mL |
| Olmesartan | 1 | 0.73% | 0.99% | 90 ng/mL | 1 μg/mL |
| Perindopril | 1 | 0.73% | 0.99% | 5 ng/mL | 50 ng/mL |
| Pyridine | 1 | 0.73% | 0.99% | 9 ng/mL | 90 ng/mL |
| Prazepam | 1 | 0.73% | 0.99% | 15 ng/mL | 150 ng/mL |
| Pregabalin | 1 | 0.73% | 0.99% | 20 ng/mL | 200 ng/mL |
| Primidone | 1 | 0.73% | 0.99% | 150 ng/mL | 2 μg/mL |
| Promazine | 1 | 0.73% | 0.99% | 0.9 ng/mL | 9 ng/mL |
| Propofol | 1 | 0.73% | 0.99% | 100 ng/mL | 1 μg/mL |
| Ramipril | 1 | 0.73% | 0.99% | 0.1 ng/mL | 1 ng/mL |
| Tamoxifen | 1 | 0.73% | 0.99% | 4 ng/mL | 40 ng/mL |
| Trazodone | 1 | 0.73% | 0.99% | 70 ng/mL | 700 ng/mL |
| Ziprasidone | 1 | 0.73% | 0.99% | 1 ng/mL | 15 ng/mL |

TABLE S3 In the table are reported the cause and manner of death, the sex, the range of age, the setting of corpse discovery, the results of toxicological analyses and the clinical history or other considerations about the case under investigation. Drug related deaths and non-drug related deaths were considered only in presence of some toxicological results; therefore, the cases with negative toxicological results, the “√” was absent. A summary about sex, average age, ethnicity, body setting discovery, total number of cases analyzed, percentage of positive results, total number of acute intoxications is reported at the end of the table

| Manner and cause of death | | | | | no. | | Toxicological analyses | | Substances detected | | Drug related death | | Non-drug related death | | |  |
| --- | --- | --- | --- | --- | --- | --- | --- | --- | --- | --- | --- | --- | --- | --- | --- | --- |
| ACCIDENTAL DEATH |  | Fall | | | | **101** | | Negative | | / | |  | |  |  |  |
|  |  |  |  |  |  | **110** | | Positive | | LSD, THC (traces) | | √ | |  |  |  |
|  |  |  |  |  |  | **120** | | Positive | | Venlafaxine | |  | | √ |  |  |
|  |  | Car accident | | | | **2** | | Positive | | Ethanol | |  | | √ |  |  |
|  |  |  |  |  |  | **7** | | Positive | | Midazolam | |  | | √ |  |  |
|  |  |  |  |  |  | **24** | | Negative | | / | |  | |  |  |  |
|  |  |  |  |  |  | **32** | | Positive | | Ethanol | |  | | √ |  |  |
|  |  |  |  |  |  | **41** | | Positive | | Ethanol | |  | | √ |  |  |
|  |  |  |  |  |  | **49** | | Negative | | / | |  | |  |  |  |
|  |  |  |  |  |  | **51** | | Negative | | / | |  | |  |  |  |
|  |  |  |  |  |  | **54** | | Negative | | / | |  | |  |  |  |
|  |  |  |  |  |  | **57** | | Negative | | / | |  | |  |  |  |
|  |  |  |  |  |  | **59** | | Negative | | / | |  | |  |  |  |
|  |  |  |  |  |  | **71** | | Positive | | Ethanol | |  | | √ |  |  |
|  |  |  |  |  |  | **74** | | Negative | | / | |  | | √ |  |  |
|  |  |  |  |  |  | **132** | | Positive | | Diazepam (traces), midazolam, ketamine | |  | | √ |  |  |
|  |  | Work accident | | | | **39** | | Negative | | / | |  | | √ |  |  |
|  | ACUTE INTOXICATION | Cocaine | | | | **3** | | Positive | | Acute intoxication | | √ | |  |  |  |
|  |  | Cocaine | | | | **4** | | Positive | | Acute intoxication | | √ | |  |  |  |
|  |  | Free morphine | | | | **8** | | Positive | | Acute intoxication | | √ | |  |  |  |
|  |  | Free morphine, cocaine | | | | **15** | | Positive | | Acute intoxication | | √ | |  |  |  |
|  |  | Cocaine, sertraline | | | | **16** | | Positive | | Acute intoxication | | √ | |  |  |  |
|  |  | Delorazepam | | | | **26** | | Positive | | Acute intoxication | | √ | |  |  |  |
|  |  | Olanzapine, paroxetine | | | | **29** | | Positive | | Acute intoxication | | √ | |  |  |  |
|  |  | Quetiapine | | | | **34** | | Positive | | Acute intoxication | | √ | |  |  |  |
|  |  | Cocaine | | | | **35** | | Positive | | Acute intoxication | | √ | |  |  |  |
|  |  | Cocaine, MDMA | | | | **36** | | Positive | | Acute intoxication | | √ | |  |  |  |
|  |  | Sertraline | | | | **37** | | Positive | | Acute intoxication | | √ | |  |  |  |
|  |  | Cocaine, free morphine | | | | **52** | | Positive | | Acute intoxication | | √ | |  |  |  |
|  |  | Cocaine | | | | **55** | | Positive | | Acute intoxication | | √ | |  |  |  |
|  |  | Free morphine | | | | **56** | | Positive | | Acute intoxication | | √ | |  |  |  |
|  |  | Oxycodone, bromazepam, lormetazepam | | | | **58** | | Positive | | Acute intoxication | | √ | |  |  |  |
|  |  | Cocaine | | | | **61** | | Positive | | Acute intoxication | | √ | |  |  |  |
|  |  | Cocaine, methadone | | | | **62** | | Positive | | Acute intoxication | | √ | |  |  |  |
|  |  | Heroin | | | | **64** | | Positive | | Acute intoxication | | √ | |  |  |  |
|  |  | Cocaine | | | | **69** | | Positive | | Acute intoxication | | √ | |  |  |  |
|  |  | Cocaine | | | | **70** | | Positive | | Acute intoxication | | √ | |  |  |  |
|  |  | Diazepam, lorazepam | | | | **73** | | Positive | | Acute intoxication | | √ | |  |  |  |
|  |  | Cocaine | | | | **75** | | Positive | | Acute intoxication | | √ | |  |  |  |
|  |  | Diazepam, bromazepam, lormetazepam | | | | **83** | | Positive | | Acute intoxication | | √ | |  |  |  |
|  |  | Cocaine | | | | **89** | | Positive | | Acute intoxication | | √ | |  |  |  |
|  |  | Cocaine | | | | **91** | | Positive | | Acute intoxication | | √ | |  |  |  |
|  |  | Methadone | | | | **93** | | Positive | | Acute intoxication | | √ | |  |  |  |
|  |  | Cocaine | | | | **96** | | Positive | | Acute intoxication | | √ | |  |  |  |
|  |  | Cocaine | | | | **97** | | Positive | | Acute intoxication | | √ | |  |  |  |
|  |  | Free morphine | | | | **98** | | Positive | | Acute intoxication | | √ | |  |  |  |
|  |  | Free morphine | | | | **99** | | Positive | | Acute intoxication | | √ | |  |  |  |
|  |  | Cocaine, paroxetine | | | | **100** | | Positive | | Acute intoxication | | √ | |  |  |  |
|  |  | Cocaine | | | | **113** | | Positive | | Acute intoxication | | √ | |  |  |  |
|  |  | Methadone | | | | **116** | | Positive | | Acute intoxication | | √ | |  |  |  |
|  |  | Cocaine, methomyl | | | | **117** | | Positive | | Acute intoxication | | √ | |  |  |  |
|  |  | Cocaine, free morphine | | | | **118** | | Positive | | Acute intoxication | | √ | |  |  |  |
|  |  | Pentobarbital | | | | **119** | | Positive | | Acute intoxication | | √ | |  |  |  |
|  |  | Alprazolam, tramadol, quetiapine, venlafaxine | | | | **121** | | Positive | | Acute intoxication | | √ | |  |  |  |
|  |  | Free morphine | | | | **123** | | Positive | | Acute intoxication | | √ | |  |  |  |
|  |  | MDPHP, cocaine | | | | **124** | | Positive | | Acute intoxication | | √ | |  |  |  |
|  |  | Cocaine | | | | **125** | | Positive | | Acute intoxication | | √ | |  |  |  |
|  |  | Ketamine | | | | **127** | | Positive | | Acute intoxication | | √ | |  |  |  |
|  |  | Free morphine, cocaine, ethanol | | | | **129** | | Positive | | Acute intoxication | | √ | |  |  |  |
|  |  | Cocaine, free morphine | | | | **130** | | Positive | | Acute intoxication | | √ | |  |  |  |
|  |  | Amlodipine, alprazolam | | | | **131** | | Positive | | Acute intoxication | | √ | |  |  |  |
|  |  | Methadone | | | | **135** | | Positive | | Acute intoxication | | √ | |  |  |  |
|  |  | Cocaine | | | | **136** | | Positive | | Acute intoxication | | √ | |  |  |  |
| SUicide | SIMPLE SUICIDE | 1 shot gun in the head | | | | **27** | | Positive | | Delorazepam, zolpidem | |  | | √ |  |  |
|  |  | Cold weapon | | | | **31** | | Positive | | Cocaine, benzoylecgonine, diazepam, EDDP, free morphine | |  | | √ |  |  |
|  |  | Hanging | | | | **17** | | Negative | | / | |  | |  |  |  |
|  |  | Suicide by fall | | | | **12** | | Positive | | Venlafaxine, zolpidem | |  | | √ |  |  |
|  |  |  |  |  |  | **46** | | Positive | | Clozapine | |  | | √ |  |  |
|  |  |  |  |  |  | **86** | | Positive | | MDPV, benzoylecgonine, cocaine | |  | | √ |  |  |
|  |  |  |  |  |  | **103** | | Positive | | Delorazepam, ziprasidone, fluoxetine, ethanol | |  | | √ |  |  |
|  |  |  |  |  |  | **134** | | Positive | | Fluoxetine | |  | | √ |  |  |
|  |  | Plastic bag suffocation | | | | **95** | | Negative | | / | |  | |  |  |  |
|  |  | Acute intoxication | Delorazepam, alprazolam | | | **11** | | Positive | | Acute intoxication | | √ | |  |  |  |
|  |  |  | Tramadol | | | **82** | | Positive | | Acute intoxication | | √ | |  |  |  |
|  |  |  | Cyanide | | | **109** | | Positive | | Acute intoxication | | √ | |  |  |  |
|  | COMPLEX SUICIDE | Acute intoxication by cocaine + GPL gas | | | | **18** | | Positive | | Acute intoxication | | √ | |  |  |  |
|  |  | Acute intoxication by butane + plastic bag suffocation (PBS) | | | | **20** | | Positive | | Acute intoxication | |  | | √ |  |  |
|  |  | Acute intoxication by cocaine + hanged | | | | **22** | | Positive | | Acute intoxication | |  | | √ |  |  |
|  |  | Acute intoxication by delorazepam and bromazepam + gas | | | | **40** | | Positive | | Acute intoxication | |  | | √ |  |  |
|  |  | Acute intoxication by clozapine (combined with other substances) + strangulation | | | | **102** | | Positive | | Acute intoxication | |  | | √ |  |  |
| Homicide |  | push down by the 6^th^ floor | | | | **81** | | Positive | | Ethanol | |  | | √ |  |  |
|  |  | More than 11 shot guns in the chest | | | | **28** | | Negative | | / | |  | |  |  |  |
|  |  | Beatings | | | | **42** | | Positive | | Ethanol | |  | | √ |  |  |
|  |  | Hit by a car | | | | **107** | | Negative | | / | |  | |  |  |  |
| natural death | HEART FAILURE |  | | | | **1** | | Positive | | Delorazepam, mirtazapine, tramadol | |  | | √ |  |  |
|  |  |  |  |  |  | **5** | | Negative | | / | |  | |  |  |  |
|  |  |  |  |  |  | **6** | | Positive | | Fentanyl, midazolam | |  | | √ |  |  |
|  |  |  |  |  |  | **9** | | Positive | | Clonazepam, THC | |  | | √ |  |  |
|  |  |  |  |  |  | **10** | | Positive | | Benzoilecgonina, cocaine, fentanyl | |  | | √ |  |  |
|  |  |  |  |  |  | **13** | | Negative | |  | | | |  |  | |
|  |  |  |  |  |  | **14** | | Positive | | Phenobarbital, diazepam, ethanol | |  | | √ |  |  |
|  |  |  |  |  |  | **19** | | Positive | | Amlodipine, ramipril, trazodone | |  | | √ |  |  |
|  |  |  |  |  |  | **21** | | Negative | | / | |  | |  |  |  |
|  |  |  |  |  |  | **23** | | Positive | | Benzoilecgonina, cocaine, cotinine | |  | | √ |  |  |
|  |  |  |  |  |  | **25** | | Positive | | Ethanol | |  | | √ |  |  |
|  |  |  |  |  |  | **30** | | Positive | | Diazepam, promazine | |  | | √ |  |  |
|  |  |  |  |  |  | **33** | | Negative | | / | |  | |  |  |  |
|  |  |  |  |  |  | **38** | | Negative | | / | |  | |  |  |  |
|  |  |  |  |  |  | **43** | | Negative | | / | |  | |  |  |  |
|  |  |  |  |  |  | **44** | | Positive | | Pyridine, tramadol, THC, paracetamol, metals (chrome and nickel) | |  | | √ |  |  |
|  |  |  |  |  |  | **45** | | Positive | | Citalopram | |  | | √ |  |  |
|  |  |  |  |  |  | **47** | | Negative | | / | |  | |  |  |  |
|  |  |  |  |  |  | **48** | | Positive | | Diazepam | |  | | √ |  |  |
|  |  |  |  |  |  | **50** | | Negative | | / | |  | |  |  |  |
|  |  |  |  |  |  | **53** | | Positive | | Amlodipine, diazepam | |  | | √ |  |  |
|  |  |  |  |  |  | **60** | | Negative | | / | |  | |  |  |  |
|  |  |  |  |  |  | **63** | | Positive | | Ethanol | |  | | √ |  |  |
|  |  |  |  |  |  | **65** | | Positive | | Lormetazepam, zolpidem | |  | | √ |  |  |
|  |  |  |  |  |  | **66** | | Positive | | Cocaine, benzoylecgonine, delorazepam, diazepam | |  | | √ |  |  |
|  |  |  |  |  |  | **67** | | Negative | | / | |  | |  |  |  |
|  |  |  |  |  |  | **68** | | Negative | | / | |  | |  |  |  |
|  |  |  |  |  |  | **72** | | Negative | | / | |  | |  |  |  |
|  |  |  |  |  |  | **76** | | Negative | | / | |  | |  |  |  |
|  |  |  |  |  |  | **77** | | Positive | | Cocaine, benzoylecgonine, nordiazepam, THCCOOH | |  | | √ |  |  |
|  |  |  |  |  |  | **78** | | Negative | | / | |  | |  |  |  |
|  |  |  |  |  |  | **79** | | Positive | | Midazolam, diazepam, ketorolac | |  | | √ |  |  |
|  |  |  |  |  |  | **80** | | Negative | | / | |  | |  |  |  |
|  |  |  |  |  |  | **84** | | Positive | | Bromazepam | |  | | √ |  |  |
|  |  |  |  |  |  | **85** | | Negative | | / | |  | |  |  |  |
|  |  |  |  |  |  | **87** | | Negative | | / | |  | |  |  |  |
|  |  |  |  |  |  | **88** | | Positive | | Fluoxetine, THC, THCCOOH | |  | | √ |  |  |
|  |  |  |  |  |  | **90** | | Negative | | / | |  | |  |  |  |
|  |  |  |  |  |  | **92** | | Positive | | Bisoprolol, Olmesartan | |  | | √ |  |  |
|  |  |  |  |  |  | **94** | | Positive | | Oxycodone | |  | | √ |  |  |
|  |  |  |  |  |  | **104** | | Positive | | Ethanol | |  | | √ |  |  |
|  |  |  |  |  |  | **105** | | Positive | | Ethanol, bromazepam, zolpidem | |  | | √ |  |  |
|  |  |  |  |  |  | **106** | | Positive | | Ethanol, bromazepam, zolpidem | |  | | √ |  |  |
|  |  |  |  |  |  | **108** | | Positive | | Alprazolam, cocaine, benzoylecgonine, allopurinol, ethanol | |  | | √ |  |  |
|  |  |  |  |  |  | **111** | | Negative | | / | |  | |  |  |  |
|  |  |  |  |  |  | **112** | | Negative | | / | |  | |  |  |  |
|  |  |  |  |  |  | **114** | | Negative | | / | |  | |  |  |  |
|  |  |  |  |  |  | **115** | | Negative | | / | |  | |  |  |  |
|  |  |  |  |  |  | **122** | | Positive | | Haloperidol, quetiapine, delorazepam, ethanol | |  | | √ |  |  |
|  |  |  |  |  |  | **126** | | Positive | | Prazepam, nordiazepam | |  | | √ |  |  |
|  |  |  |  |  |  | **128** | | Positive | | Benzoylecgonine, cocaine,  zolpidem, cotinine, ethanol | |  | | √ |  |  |
|  |  |  |  |  |  | **133** | | Positive | | Benzoylecgonine, cocaine, propofol | |  | | √ |  |  |
| Sex | | | | Males: 103 subjects; Females: 33 individuals | | | | | | | | | | | |  |
| Average age | | | | 41-50 range of age for both genders | | | | | | | | | | | |  |
| ETHNICITY | | | | Caucasian (131 cases); Latin American (2 subjects); Asian (2 cases); African (1 subject) | | | | | | | | | | | |  |
| Body setting discovery | | | | House (34); hotel (5); park (4); Car (4); hospital (4); Jail (2); airport (1); work (1); train (1); street (1); swimming pool (1); | | | | | | | | | | | |  |
| total number of cases analyzed | | | | 136 | | | | | | | | | | | |  |
| % of positive case | | | | 74.3% (101 cases) | | | | | | | | | | | |  |
| total number of acute intoxication | | | | 54 cases (46 unvoluntary lethal acute intoxication; 3 simple suicides by substances; 5 complex suicides) | | | | | | | | | | | |  |
